# Cyclosporine-a attenuates retinal inflammation by inhibiting HMGB-1 formation in rats with type 2 diabetes mellitus

**DOI:** 10.1186/s40360-020-0387-6

**Published:** 2020-02-04

**Authors:** Peng Wang, Fei Chen, Xuedong Zhang

**Affiliations:** Department of Ophthalmology, Chongqing Key Laboratory of Ophthalmology, Chongqing Eye Institute, the First Affiliated Hospital of Chongqing Medical University, No.1 You Yi Road, Yu Zhong District, Chongqing, 400016 China

**Keywords:** Cyclosporine-a, Diabetic retinopathy, HMGB-1, Inflammation, IL-1β, TNF-α

## Abstract

**Background:**

Cyclosporine-A has been regarded as an immunoregulatory and anti-inflammatory drug for the treatment of various immune inflammatory diseases. However, the effect of Cyclosporine-A on the retina of type 2 diabetic rats and the underlying mechanism remains to be elucidated. The objective of the present study was to investigate the effect and mechanism of Cyclosporine-A on diabetic retinopathy.

**Methods:**

Male Sprague-Dawley rats were established to type 2 diabetic model. After 6 weeks, diabetic rats and normal controls were intravitreally injected with. Cs-A (42 ng/2 μL) to the left eye, and 2 μL DMSO to the right eye for the control.. Another group of normal wild-type rats was subjected to intravitreal injections into. The left eyes with 5 μL PBS or HMGB-1 (5 ng/5 μL) or HMGB-1(5 ng/5 μL) plus. Cs-A (42 ng/2 μL), respectively. Retinal morphological changes were observed with. Hematoxylin–eosin staining. Expressions of HMGB-1, IL-1β and TNF-α were. Detected by immunohistochemistry, ELISA or Western blot or RT-PCR.

**Results:**

Retinal expression levels of IL-1β and TNF-α were upregulated in type 2. diabetic rats and in normal rats with intravitreal injection of HMGB-1, which were. Attenuated by intravitreal Cs-A. Moreover, Cs-A decreased HMGB-1 expression in. diabetic retina and relieved the retinopathy in type 2 diabetic rats.

**Conclusions:**

Intravitreal administration of Cs-A showed a protective effect on retina. of diabetic rats, possibly by downregulating retinal expressions of IL-1β and TNF-α. via the suppression of HMGB-1.

## Background

Diabetic retinopathy (DR) is the most common ocular complication of diabetes mellitus (DM), occurring in more than 60% of patients with type 2 DM [1]. Along. with the increasing prevalence of DM worldwide [2], DR is the leading cause of visual impairment and blindness in adults, which results in heavy economic burdens for healthcare systems. The underlying mechanism of DR is intricate and remains incompletely revealed. Mounting evidence suggests that chronic low-grade inflammation is involved in the pathogenesis of DR [3]. In the early stages of DR, expression levels of inflammatory cytokines, namely interleukin-1β (IL-1β) and tumor necrosis factor-α (TNF-α), are elevated in the retina, vitreous and serum of diabetic patients and rodents [4, 5]. Those cytokines are believed to promote leucocyte adhesion and vascular lesions in the retina. In addition, recent studies indicate that high mobility group box-1 (HMGB-1) protein, a proinflammatory. Mediator, participates in DR via activation of inflammatory cascades [6, 7]. Cyclosporine-A (Cs-A) was firstly used to prevent organ rejection after clinical. Transplantation as a potent immunosuppressant [8]. Later, Cs-A has been regarded as. an immunoregulatory and anti-inflammatory drug for the treatment of various. Immune inflammatory diseases, such as Bechet’s syndrome [9] and atopic keratoconjunctivitis [10]. Moreover, a few studies show that Cs-A has a protective effect on the retina of streptozotocin (STZ)-induced diabetic rats, possibly through impeding the immunoglobulins deposition [11] or reducing the blood-retinal barrier permeability [12] in the diabetic retina. However, little has been known about the impact of Cs-A on the retina of type 2 diabetic rats and the underlying mechanism. Therefore, the aim of our study was to investigate the effect of Cs-A on the retinopathy in a type 2 DM animal model and to elucidate its potential mechanism, specifically, to study the alternation of expression levels of HMGB-1, IL-1β and TNF-α in the retinal tissues.

## Methods

### Animals

Healthy male Sprague-Dawley rats (*n* = 64, aged 8-10 weeks, weighed between 200 and 250 g) were used for all experiments. All rats were purchased from the. Experimental Animal Center of Chongqing Medical University, where rats were. Housed in cages on a 12-h light-dark schedule with food and water ad libitum. All procedures were performed in accordance to the Chongqing Medical University’s Animal Care and Use Committee Guidelines.

### Induction of type 2 diabetes mellitus

After 1 week of adaptive feeding, 40 rats were randomly divided into two groups: Normal group and Diabetic group. Rats in the Normal group were fed with normal chow diet, while those in the DM group with a high-fat and high-glucose diet. Four weeks later, streptozotocin (STZ; Sigma-Aldrich) was prepared in acetate buffer and administered (30 mg/kg) via intraperitoneal injections for 5 consecutive days at 7–9 weeks of age. Normal group mice were administered an intraperitoneal injection of acetate buffer (pH 4.5). After injection, blood glucose level greater than 16.7 mmol/L was taken as the successful establishment of type 2 DM model. After successful diabetes induction, rats were treated according to the study design. At the end of the treatment, animals were sacrificed with overdose pentobarbital. Rat eyeballs were enucleated and immediately put into 40 g/L paraformaldehyde for hematoxylin and eosin (H-E) staining or immunohistochemistry. Retinas were isolated carefully and snap frozen in liquid nitrogen for western blot or enzyme-linked immunosorbent assay (ELISA).

### Intravitreal treatment of Cs-a/HMGB-1

This study comprised of two experimental parts. First, rats in the Normal and DM group were deeply anesthetized by pentobarbital injection, and topical ocular anesthesia was achieved with 0.4% benoxinate hydrochloride of eye drop. After pupils were dilated satisfactorily, rats were injected with Cs-A (42 ng/2 μL Dimethyl sulfoxide (DMSO)) intravitreally in the left eye with a 30-gauge micro-injector under a dissecting microscope. For the control, 2 μL DMSO was injected into the right eye of the same rat. According to the treatment, rat eyes could be categorized into four groups: Normal, Normal+Cs-A, DM and DM + Cs-A group. Rats with operational complications such as vitreous hemorrhage, retinal detachment or death were excluded from our study. Forty-eight hours after administration of Cs-A, rats were anesthetized and sacrificed for further experiments.

The second part of the experiment was that twenty-four normal healthy rats. (Sprague-Dawley, non-diabetic, 200–250 g) were subjected to intravitreal injections into the left eyes using the method described above. Rats were divided into three groups according to the injection substances: Normal control group (*n* = 8) received 5 μL sterile phosphate buffer saline (PBS); Normal+HMGB-1 group (*n* = 8) received sterilized solution of recombinant HMGB-1 (5 ng/5 μL PBS; R&D Systems, Minneapolis, MN); and Normal+HMGB-1 + Cs-A group (*n* = 8) received a combination of HMGB-1(5 ng/5 μL PBS) and Cs-A (42 ng/2 μL DMSO).

### H-E staining of the retinal tissues

Rat eyeballs were dehydrated using graded ethanol and embedded in paraffn. Sections of five-micron thickness were cut and transferred to triethoxysilane-coated slides.

The tissues were stained with hematoxylin–eosin and examined for morphometry. Images were taken through an Olympus BX60 microscope (Olympus Optical Co Ltd., Tokyo, Japan).

### Immunohistochemistry and Western blot

For immunohistochemistry, the paraffin-embedded retinal sections (5 μm) were dewaxed and dehydrated. Endogenous peroxidase was quenched with 3% H_2_O_2_ blocker for 10 min at room temperature. Then, the sections were incubated with anti-HMGB-1 antibody (1:500, Epitomics, Cambridge, U.K.) at 4 °C overnight. After extensive washing with PBS, the sections were incubated with horseradish peroxidase -streptavidin immunoglobulin (1:500, Abcam, Cambridge, UK) for 30 min, developed with diaminobenzidine for 3 min and counterstained with hematoxylin. Quantitative image analysis was performed with Image-Pro Plus 6.0 software (Media Cybernetics Inc., Bethesda, Maryland MD, USA). Ten fields per retina were randomly selected and the densitometry mean values of HMGB-1 immunostaining were determined.

For western blot, protein extracts were collected with a tissue lysis buffer (PBS containing 10 mM EDTA, 1% Triton X-100 and the protease inhibitor cocktail) and the concentrations were determined using a bicinchoninic acid protein assay kit (Bio-Rad, Richmond, CA). Protein samples were separated on polyacrylamide gel electrophoresis and transferred to a polyvinylidene difluoride membrane. The membrane was blocked with 5% dry milk in Tris-buffered saline with 0.1% Tween 20 (TBST) for 1 h. Primary antibodies were incubated at 4 °C overnight at the following dilutions: anti-HMGB-1 antibody (1:500, Epitomics, Cambridge, U.K.), anti- IL-1β antibody (1:500,Epitomics, Cambridge, U.K), anti-TNF-α antibody (1:500, Abcam, Cambridge, U.K) or anti-actin antibody (1:1000, Sigma, St. Louis, MO, USA), and then incubated with secondary horseradish peroxidase-conjugated antibody (1:1000, Abcam, Cambridge, U.K.) for 1 h at 37 °C. The immunocomplexes were visualized by the ECL chemiluminescence method. Subsequently, semi-quantitative analysis was performed using the quantity one software (Bio-Rad, Richmond, CA). The amount of target proteins was quantified relative to the level of β-actin.

### ELISA assay of IL-1β and TNF-α

At the end of the treatment, each retina was dissected and homogenized in 100 μL of lysis buffer supplemented with protease inhibitors (Beyotime). Samples were centrifuged at a speed of 12,000 rpm for 10 min at 4 °C and the supernatants were collected. After protein concentrations were assessed with the Bio-Rad method (Bio-Rad, Richmond, CA), samples were subjected to corresponding ELISA kits (R&D Systems, Minneapolis, MN) for the determination of IL-1β and TNF-α levels according to the manufacturer’s instructions. The absorbance at 450 nm was read on an automated plate reader (Spectra Max Gemini UVmax; Molecular Devices, Sunnyvale, CA). All measurements were performed in triplicate and the tissue sample concentration was calculated from a stand curve and corrected for protein concentration.

### RNA extraction and RT-PCR

The retinas were separated from eyeballs. Total RNA was extracted from mouse retinal tissues using a RNAiso kit (Invitrogen, Paisley, UK) according to the manufacturer’s instructions. Reverse transcription was carried out on the extracted total RNA by a reverse transcription kit (Mbi, Glen Burnie, MD, USA) to obtain cDNA, and the operation steps were carried out according to the reverse transcription kit manufacturer’s instructions. Amplifcation was carried out by SYBR Green kit (Roche Diagnostics, Basel, Switzerland), and IL-1β primer, TNF-α primer and GAPDH primer were synthesized and designed by Shanghai Sangon Biological Engineering Technology & Services Corporation. More details are shown in Table 1. The PCR amplifcation conditions were as follows: pre-incubation for 5 min at 94 °C, 35 cycles of amplification (94 °C for 30 s, 55 °C for 30 s, and 72 °C for 30 s). Both IL-1β and TNF-α were normalized to GAPDH expression using the 2^-ΔΔCt^ method.

**Table 1:** Primer sequence

Gene	Upstream primer	Downstream primer
IL-1β	3′-TGGCAATGAGGATGACTTGT-5	3′-TGGTGGTCGGAGATTCGTA-5′
TNF-α	3′-GAGCACTGAAAGCATGATCC-5′	3′-CGAGAAGATGATCTGACTGCC-5′
GAPDH	3′-CTAGACCCAGTAGAAGAGCG-5′	3′-GATAGGTCCGCAACGATAGG-5′

### Statistical analysis

The data were expressed as mean ± standard deviation (SD). Data were analyzed using the one-way analysis of variance (ANOVA) followed by Bonferroni’s post hoc test. All statistical analyses were performed with GraphPad Prism software (version 4.0, GraphPad Software, San Diego, Calif.). *P* value less than 0.05 was considered statistically significant.

## Results

### Animal characteristics

At the end of the experiment period, the fasting blood glucose levels of rats in the DM group were significantly higher than those in the Normal group (16.81 ± 3.14 vs. 5.04 ± 0.48 mmol/L, *p < 0.01*). However, there was no significant difference in fasting blood glucose levels between DM and DM + Cs-A group (16.81 ± 3.14 vs. 15.04 ± 4.28 mmol/L), Normal and Normal +Cs-A group (5.04 ± 0.48 vs. 5.81 ± 0.73 mmol/L), respectively.

### The pathomorphological changes of retinal tissues

**Fig. 1:**
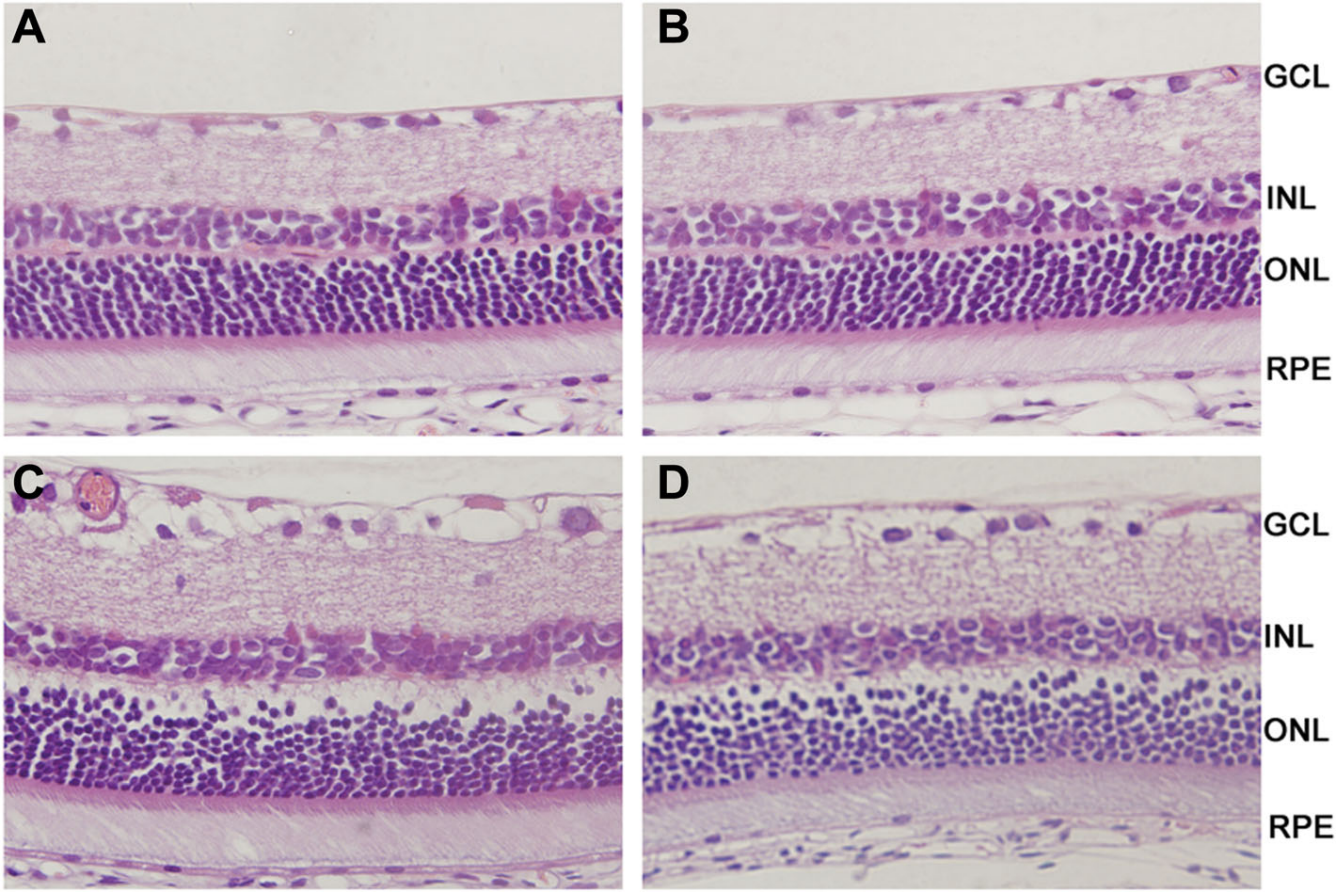
Photomicrographs of rat retinas with H-E staining: (**a**) Normal group: retinal cells displayed normal shapes and orderly arrangements in every layer. of the retinal tissues; (**b**) Normal +Cs-A group: no obvious abnormalities were. observed compared to the normal group; (**c**) DM group: there were edema and. thickening of ganglion cell layer, inner plexiform layer and outer nuclear layer, besides disordered alignments of inner nuclear layer; (**d**) DM + Cs-A group: there were approximately regular arrangements of retinal cells and slightly thickened retina tissues. Original magnification was 400X.

Retinal tissues with HE staining showed no remarkable retinal abnormalities in the Normal and Normal +Cs-A group, as retinal cells were well-shaped and organically-aligned in each layer of the retinal tissues (Fig. 1a and b). In the DM group, there was presence of thickening and edema in the retinal ganglion cell layer, inner plexiform layer, and outer nuclear layer, with disordered retinal cell arrangements especially in the inner nuclear layer and outer nuclear layer (Fig. 1c). On the contrary, abnormalities of cell edema and disarrangement were obviously attenuated in the DM + Cs-A group (Fig. 1d).

### Immunohistochemical detection and the protein expression of HMGB-1

**Fig. 2:**
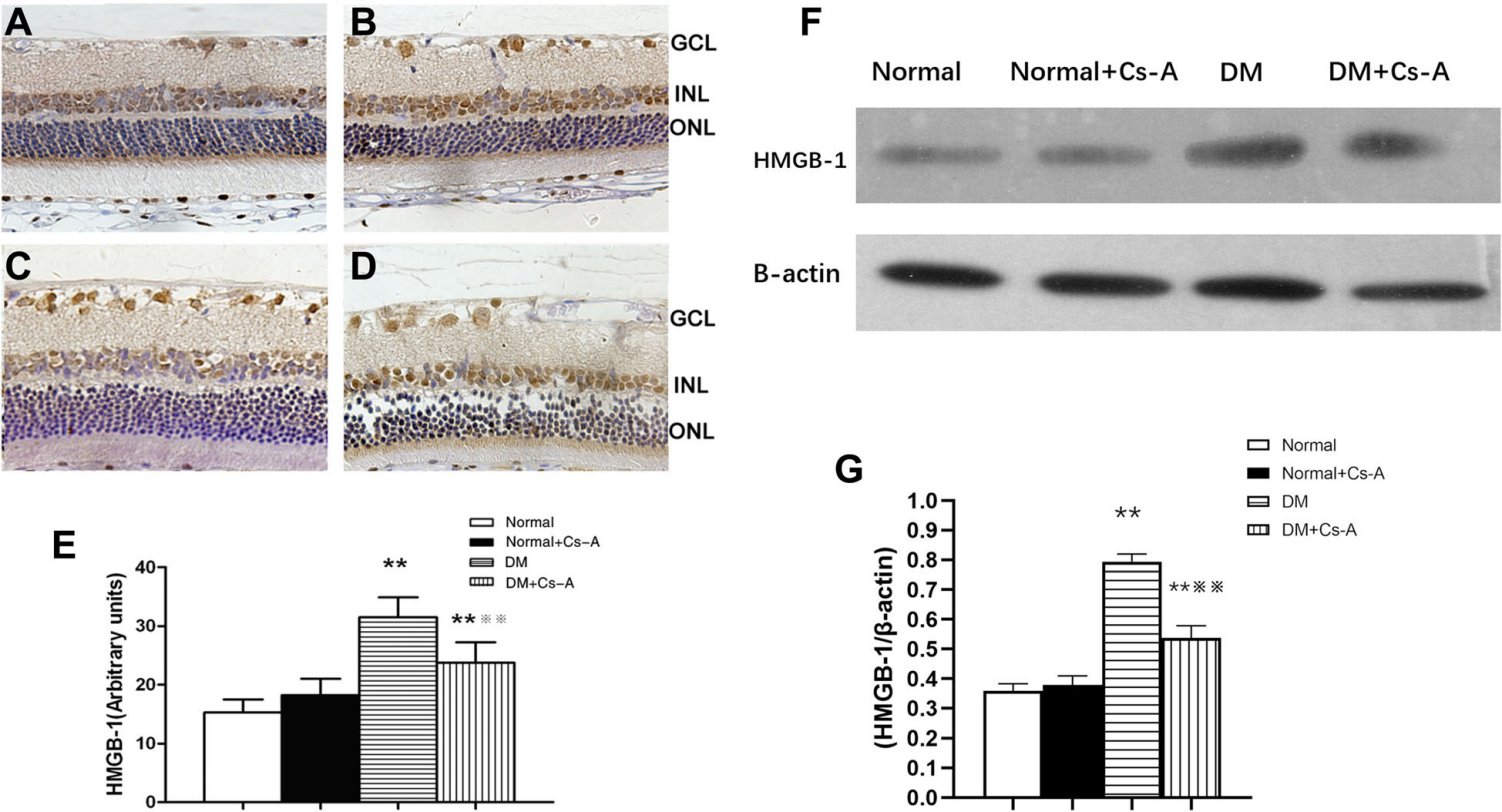
Immunohistochemical detection and the protein expression of HMGB-1 (**a**)Normal group (**b**) Normal +Cs-A group (**c**) DM group and (**d**) DM + Cs-A group. Original magnification was 400X. Semiquantitative analyses of HMGB-1 levels were shown in (**e**). Data are mean ± SD, **p < 0.01 vs. Normal group and Normal +Cs-A group, and ※※*P < 0.01* vs. DM group. (**f**) The expression HMGB-1 protein in Normal, Normal+Cs-A, DM and DM + Cs-A group respectively. (**g**) Mean ± SD of HMGB-1 protein level normalized to β-actin (internal control) were calculated. **p < 0.01 vs. Normal group and Normal +Cs-A group, and ※※*p < 0.01* vs. DM group

Location and expression level of HMGB-1 in the retinal tissues were determined by immunohistochemistry and western blot methods. The levels of immunostaining to HMGB-1 in retinas were significantly higher in the diabetic rats than in the normal ones (Fig. 2a and c), and Cs-A treatment significantly reduced this effect induced by diabetes (Fig. 2d). However, Cs-A treatment had no obvious effect on HMGB-1 expression in the retinas of normal rats (Fig. 2a and b).

Retinal HMGB-1 protein expression was significantly higher in the diabetic rats than in the normal ones (Fig. 2f), and Cs-A treatment significantly reduced this effect induced by diabetes (Fig. 2f and g).

### Retinal protein and mRNA expressions of IL-1β and TNF-α with Cs-a treatment

**Fig. 3:**
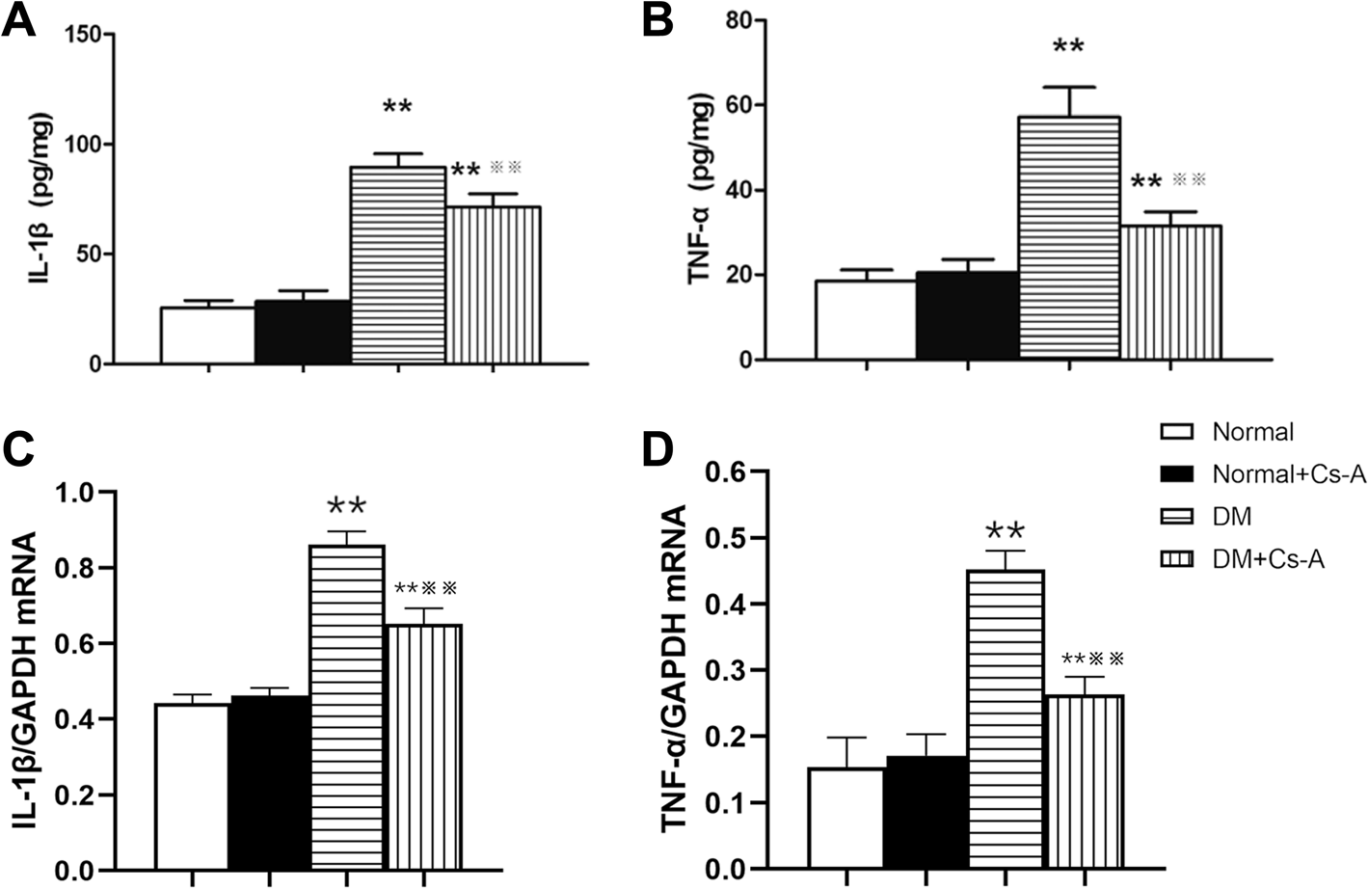
Retinal protein and mRNA expressions of IL-1β and TNF-α with Cs-A treatment: ELISA assay and Western Blot were performed to determine the retinal protein and mRNA expressions of IL-1β and TNF-α in the Normal, Normal +Cs-A, DM and DM + Cs-A group. (**a**) Protein expression of IL-1β in the retina. (**b**) Protein expression of TNF-α in the retina. (**c**) mRNA expression of IL-1β in the retina. (**d**) mRNA expression of TNF-α in the retina. Data are mean ± SD, **p < 0.01 vs. Normal group and Normal +Cs-A group, and ※※*p < 0.01* vs. DM group. (pg/mg: pg per mg of retina)

Compared with the Normal group, retinal protein and mRNA expression of IL-1β in the DM and DM + Cs-A group increased significantly (*p < 0.01*). However, the expression level of IL-1β in the DM + Cs-A group was significantly lower than that in the DM group (*p < 0.01*). There was no significant difference between the Normal and Normal +Cs-A group in retinal IL-1β expression (Fig. 3a and c). Similar trends were observed in the retinal protein expression of TNF-α (Fig. 3b and d).

### Retinal protein expressions of IL-1β and TNF-α with HMGB-1 treatment

**Fig. 4:**
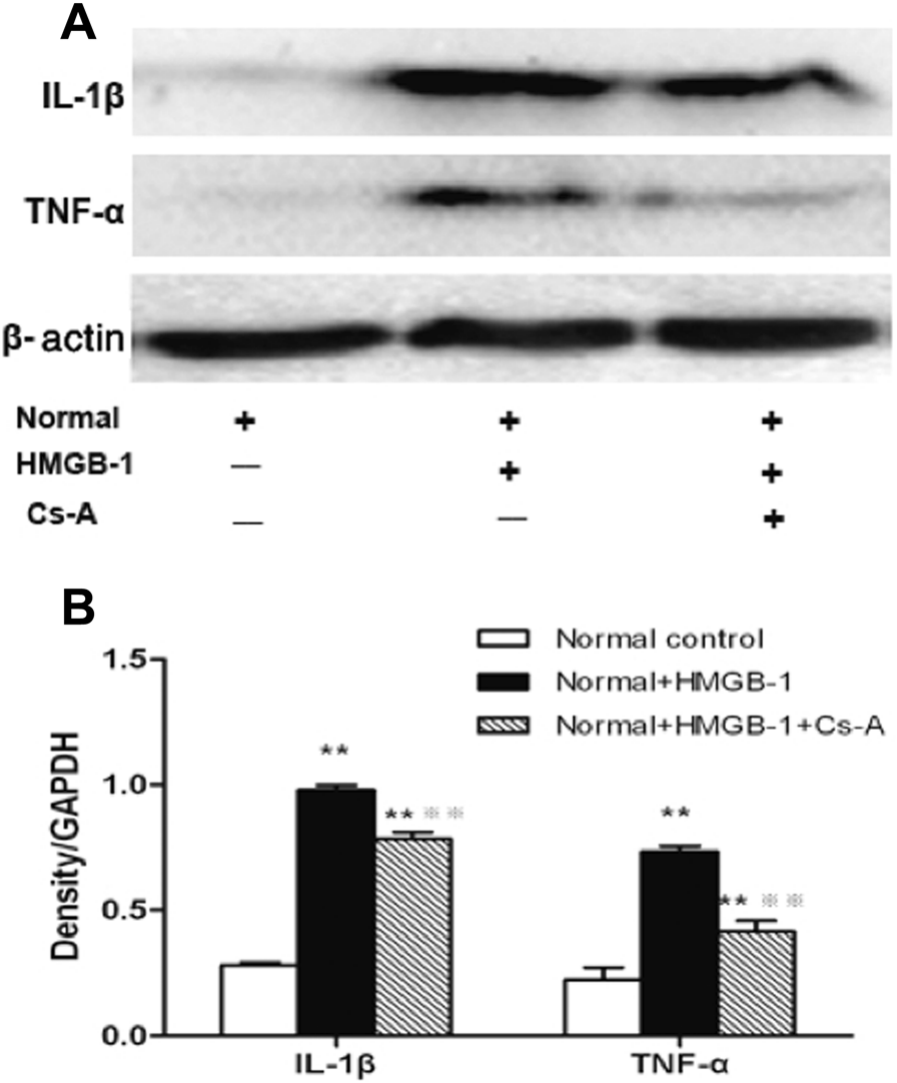
Retinal protein levels of IL-1β and TNF-α with HMGB-1 treatment. Western blot was performed to determine the retinal protein expression of. IL-1β and TNF-α in the Normal control, Normal+HMGB-1 and Normal+HMGB-1 + Cs-A group. (**a**) Representative protein immunoblots of IL-1β and TNF-α in retinal tissue. Equal amounts of proteins were loaded and β-actin was used as a loading control, *n* = 4 experiments. (**b**) Column diagrams and bars representing the ratio of the scanned immunoblots of IL-1β and TNF-α to that of β-actin, respectively. Data are mean ± SD, n = 4 experiments, **p < 0.01 vs. Normal control group, and ※※*p < 0.01* vs. Normal+HMGB-1 group.

Compared with the Normal control group, retinal protein expression of IL-1β and TNF-α in the Normal+HMGB-1 group and Normal+ HMGB-1+ Cs-A group increased significantly (*p < 0.01*, respectively). However, the expression levels of IL-1β and TNF-α in the Normal+ HMGB-1 + Cs-A group was significantly lower than that in the Normal+HMGB-1 group (*p < 0.01*, respectively) (Fig. 4).

## Discussion

Previously we have demonstrated that Cs-A has a protective effect on the structure and function of retina in rats with STZ-induced DM [13]. In the present study, we showed that Cs-A could attenuate retinal edema in diabetes-caused retinopathy, using a well-established animal model of type 2 DM by administration of a high-fat and high-glucose diet combined with a small dose of STZ injection [14]. In addition, the effect of Cs-A could be possibly attributed to the decreased expression levels of HMGB-1 and relating inflammatory mediators (IL-1β and TNF-α) in the retina. In the past decades, increasing studies have indicated that inflammation play a key role in the pathogenesis of diabetic retinopathy [3, 15–17]. There are many features typical of inflammation in the retina of diabetic patients and rodents, such as increased blood flow and vascular permeability [17], enhanced leukocyte adhesion and macrophage infiltration [18, 19], and strengthened expression of various inflammatory mediators [15, 20]. Many of those mediators have become research spots as they may stand as potential therapeutic targets for the treatment of diabetic retinopathy, IL-1β and TNF-α should be counted. The two cytokines have caused special attention for that they contribute to the development of retinopathy as well as provide neurotrophic functions to support retinal cell survival [21].

Demircan et al. [22] found that expression levels of IL-1β and TNF-α were increased in the vitreous humor and serum of patients with proliferative diabetic retinopathy. Kowluru et al. [23] and Behl et al. [24] documented that diabetes enhanced the production of IL-1β and TNF-α in the rat retina, respectively. More importantly, drug inhibition of IL-1β or gene knockout of the receptor for IL-1β significantly prevented the degeneration of retinal capillaries caused by diabetes [25]. Specific inhibitor or genetic deficiency of TNF-α greatly reduced the leukocyte adhesion and blood-retinal barrier breakdown in the diabetic retina [5, 24]. In agreement with these studies, we demonstrated a significant upregulation of IL-1β and TNF-α in the six-week diabetic rat retina, and that intravitreal administration of Cs-A significantly decreased this upregulation induced by diabetes.

Cs-A binds to cyclophilin A, forming a drug protein complex, which blocks calcineurin (Ca2+/calmodulin- dependent protein phosphatase), and subsequently leads to the downregulation of numerous proinflammatory factors such as IL-1β and TNF-α [26]. In our study, Cs-A exerted anti-inflammatory and inhibitory effect on retinopathy in the diabetic retina via reducing the retinal levels of IL-1β and TNF-α. This result agrees with previous study reporting that Cs-A inhibits the in vivo synthesis of IL-1β and TNF-α in some thymic mice [27]. Similarly, an inhibitory effect of Cs-A was showed in vitro, for instance, in rat renal mesangial cells and in unseparated peripheral blood mononuclear cells [28, 29].

In the present study, increased HMGB-1 immunoreactivity was observed in the retina of type 2 diabetic rats compared to the normal rats. HMGB-1, originally identified as a non-histone chromatin-binding protein [30] can be released into the extracellular space either passively or actively as a signal to trigger inflammation [31]. Extracellular HMGB-1 may signal via the receptor for advanced glycation end products (RAGE) and toll-like receptors 2 and 4. Activation of these receptors results in the activation of nuclear transcription factor Kappa B (NF-ĸB), which accelerates the production of cytokines such as IL-1β and TNF-α, thereby promoting inflammation [16]. Under diabetic environment, HMGB-1 acts as a proinflammatory cytokine participating in the pathogenesis of diabetic retinopathy [6].

Moreover, Mohammad et al. [7] showed that intravitreal administration of HMGB-1 to normal rats caused “diabetic -like” retinopathy, which could be greatly relieved by a specific inhibitor of HMGB-1 (Glycyrrhizin). Consistently, we proved that intravitreal injection of HMGB-1 to normal rats upregulated the retinal levels of inflammatory mediators namely IL-1β and TNF-α, and the effect was significantly attenuated by Cs-A treatment. Our results are consistent with the in vitro study reported by Gabryel et al., which provides evidence that Cs-A decreases HMGB-1 expression in ischemic astrocytes and thus attenuates the ischemia induced necrosis and neuroinflammation [32]. Noteworthily in the present study, Cs-A did dramatically extenuate but not extinguish the adverse effect of diabetes or HMGB-1 on the rat retina. Inadequate concentration of Cs-A may be counted as a reason. Since we have previously demonstrated that Cs-A of 42 ng/2 μl were effective to ease retinopathy in STZ-induced diabetic rats [13], the type 2 diabetic rat model used in our study may also, be taken into consideration.

## Conclusions

In summary, this study shows that protein expressions of IL-1β and TNF-α were enhanced in the retina of diabetic rats and the Cs-A treatment was able to attenuate the enhancement, possibly via the suppression of HMGB-1. Altogether, these results indicate that intravitreal injection of Cs-A may represent a novel therapeutic strategy for the treatment of diabetic retinopathy. However, further studies are warranted to elucidate the molecular mechanism underlying the protective effect of Cs-A on diabetic retina.

## Data Availability

All data generated or analyzed during the present study are included in this published article.More details are available from the corresponding author on reasonable request.

## Abbreviations

Cs-A: Cyclosporine-a
DM: Diabetes mellitus
DMSO: Dimethyl sulfoxide
DR: Diabetic retinopathy
HMGB-1: High mobility group box-1
PBS: Phosphate buffer saline
RAGE: Receptor for advanced glycation end products
TNF-α: Tumor necrosis factor-α
